# Memory trace replay: the shaping of memory consolidation by neuromodulation

**DOI:** 10.1016/j.tins.2015.07.004

**Published:** 2015-09

**Authors:** Laura A. Atherton, David Dupret, Jack R. Mellor

**Affiliations:** 1 School of Physiology and Pharmacology, University of Bristol, Bristol, BS8 1TD, UK; 2 Medical Research Council Brain Network Dynamics Unit at the University of Oxford, Department of Pharmacology, Oxford, OX1 3TH, UK

**Keywords:** hippocampus, sharp-wave ripples, replay, synaptic plasticity, dopamine, acetylcholine

## Abstract

The consolidation of memories for places and events is thought to rely, at the network level, on the replay of spatially tuned neuronal firing patterns representing discrete places and spatial trajectories. This occurs in the hippocampal-entorhinal circuit during sharp wave ripple events (SWRs) that occur during sleep or rest. Here, we review theoretical models of lingering place cell excitability and behaviorally induced synaptic plasticity within cell assemblies to explain which sequences or places are replayed. We further provide new insights into how fluctuations in cholinergic tone during different behavioral states might shape the direction of replay and how dopaminergic release in response to novelty or reward can modulate which cell assemblies are replayed.

## Highlights

• Memory trace replay results from lingering excitability and synaptic plasticity.
• The balance of replay mechanisms may be determined by neuromodulation.
• Acetylcholine release can shape the direction of replay in sharp wave ripples.
• Dopamine release can dictate which cell assemblies are replayed.

## What is memory trace replay?

What determines which memories are retained and which are lost is an absorbing topic for scientists and nonscientists alike, yet the mechanisms underlying the persistence of some pieces of information and the forgetting of others remain to be identified. Well-established theories propose that memories are encoded during wake behavior, with information being represented in the coordinated activity of subsets of neurons forming cell assemblies 1, 2, 3, 4. However newly encoded memories are typically fragile and, because they may decay, require additional maintenance processes. At the network level, one such process is the off-line reactivation of assembly firing patterns observed during active behavior. This process is best illustrated by the location-specific firing of principal cells 5, 6, 7, 8 in the hippocampus. These place cells (see Glossary) are activated sequentially as an animal runs through an arena. Subsequently, co-active place cells representing a discrete place or sequential place cell activation representing a trajectory are reactivated or replayed during SWRs (Box 1, Box 2), which intermittently occur in slow wave sleep (SWS) (sSWRs), long periods of awake immobility (iSWRs), or brief pauses in exploration (eSWRs) (reviewed in 9, 10).

Sequential replay can occur in both a forward 11, 12, 13, 14, 15 and backward 11, 13, 14, 15, 16 direction, with the directional balance proposed to be dependent on the ongoing behavioral state of the animal [17]. Similar to theta-phase precession [18], these replayed sequences are temporally compressed compared with those observed during wake behavior 12, 13, 19, 20. Consequently they have been posited to provide the appropriate temporal neural activity for the Hebbian synaptic modification occurring downstream in neocortical networks during memory consolidation 1, 12, 21, 22. Although direct support for this hypothesis is currently lacking, intact NMDA receptor (NMDAR) activity during learning and intrahippocampal synaptic transmission during consolidation are at least necessary for unimpaired memory consolidation and place cell reactivation during SWRs 23, 24. Moreover, evidence suggests the hippocampus and neocortex are actively engaged during SWRs and SWS. Cortical and hippocampal sequences, reflecting the same experiences, replay together during SWS [20] and, during SWRs, prefrontal neurons consistently fire within tight temporal windows <100 ms after hippocampal pyramidal cells, which could plausibly drive plasticity at the level of single cell pairs [25].

Concordantly, a growing body of literature has linked SWRs to learning and memory. SWR incidence during SWS is increased following training on a place-reward association task [26]. Conversely, electrically interrupting SWRs during post-training sleep impaired spatial learning 27, 28, while interrupting SWRs during training on a spatial alternation task selectively impaired spatial working memory, but not spatial reference memory [29]. Critically, it remains to be determined whether the ongoing hippocampal network activity during SWRs (i.e., global transient changes in interneuron and pyramidal cell activities) or specifically the reactivation or replay of place cell activity during SWRs is the more important for spatial learning and memory. Indeed, SWR activity content can be biased towards newly learnt firing patterns and predict memory performance [24]. Moreover, recent work also showed the induction of an artificial place-preference behavior following intracranial stimulation, triggered by single-place cell activity during sleep. This further suggests that replay of place cell activity serves an important role in spatial memory [30], although how this applies to the coordinated neuronal ensemble activity during SWRs remains to be investigated.

By contrast, replay, particularly during awake SWRs, has also been proposed to have functional roles other than for spatial memory consolidation; for example, in temporal credit assignment to reward locations (particularly backward replay) 16, 31; formation of goal-relevant or novel environment place-related assemblies 24, 32, 33, 34; evaluation of trajectory choices for decision making on spatial working memory tasks for prospection and planning (particularly forward replay) 29, 35, 36, 37, 38; and representation of unexplored trajectories 15, 39. Preplay of trajectories yet to be experienced has been proposed to facilitate, at least in part, the selection of subsequent place cell representations in a novel environment [40].

Despite over a decade's worth of literature describing hippocampal replay during SWRs, several questions remain. How is neuronal coordination during SWRs controlled? What selects which trajectories will be replayed within a given SWR? Do these mechanisms differ for SWRs in different behavioral states, or under different neuromodulators? In this review, we provide a critique of the evidence surrounding two current theories on how replay occurs, namely by lingering place-related excitability or as a result of synaptic plasticity. Given the limitations of both models, we then outline how neuromodulatory factors are likely to influence the mechanisms underlying replay and impart selection onto which trajectories are replayed. Finally, we propose an integrated model for replay in SWRs that takes into account the behavioral state of the animal and the underlying neuromodulatory tone.

## Replay by lingering place-related excitability

**Figure 1:**
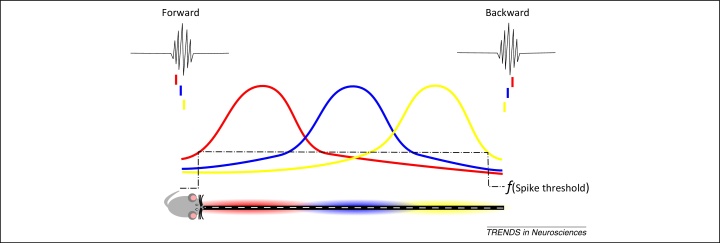
Lingering excitability model. The firing rate of a place cell (colored distributions) can be considered as a symmetrical distribution centered on the middle of the place field of the cell 41, 83. Normal spike thresholds along the track mean that the firing of each cell is turned on and then off as an animal traverses the place field of the cell. However, at the end of the track, where sharp wave ripples (SWRs) may occur as the animal slows down, the hippocampal network moves into a state where inhibitory inputs impinging onto pyramidal cells are temporally redistributed; inhibition at the axon initial segment is removed, effectively reducing the spike threshold of pyramidal cells compared with waking periods outside of SWRs [127]. This then reveals the tails of the firing distributions of the spatially tuned cells so that, during SWRs, the cells fire in an order dictated by these distributions (i.e., forward if the animal was at the start of the trajectory sequence but in reverse if the animal was at the end of the trajectory sequence).

Replay in a forward or backward direction during awake iSWRs at the ends of linear tracks and in a reverse direction during eSWRs in an open-field environment has been proposed to occur via a residual, place-selective, spatial tuning drive 11, 14, 16. In this model, place cells receive subthreshold inputs as a function of the distance of the animal from the place field center of each cell 41, 42. During SWRs, pyramidal cells have a higher firing probability and their waking patterns are reactivated 43, 44, 45. However, the replay firing content *per se* is lingered in an order dictated by the subthreshold spatial inputs onto place cells at the current position of the animal (Figure 1). This effectively represents a nonassociative bias that can influence the spontaneous SWR response of hippocampal cell assemblies [46]. Concordantly, on a linear track, there is a preference for reverse replay at the end of the track following even the first lap [16], while there is a preference for forward replay at the start of the track in anticipation of the run [14].

In support of this model, the firing probability of place cells in eSWRs increased the closer the animal was to the place field center, suggesting that the momentary, place-related, excitatory drive directly contributes to reverse reactivation in an open-field environment [11]. This is consistent with a large proportion of awake replays starting from the current location in a maze, where the spatial inputs would be stronger 13, 37, 47. Therefore, the model predicts that sequential activation of place cells in awake SWRs should not only reflect the actual path taken or future path from the current position, but also the cascades of spatially tuned activities dictated by the hippocampal map representation of the entire environment. In line with this suggestion, forward reactivation during eSWRs in a 2D open-field environment was not anticipatory to future path taken [11]; reactivation in SWRs on a spatial alternation task were equally representative of actual and alternative past–future paths [35]; and replay initiated from current location on a long linear track was not biased towards future and past trajectories [13]. However, when the task is goal driven, trajectory sequences in awake SWRs strongly represent the path to the future goal location [37].

Evidence that challenges this model comes from *in vitro* studies showing that somatic depolarization does not dramatically increase pyramidal cell spiking in SWRs 48, 49 and that there was no difference in resting membrane potential between the pyramidal cells that spike during SWRs and those that do not [48]. However, this potentially did not account for the role of synaptic inhibition, which can act to hyperpolarize the membrane (at membrane potentials above the inhibitory reversal potential) or as a shunt at the inhibitory reversal potential. Pyramidal cell spiking is dampened during SWRs by strong perisomatic inhibition 48, 50, 51, likely from parvalbumin positive basket cells, which are strongly active in SWRs 45, 52. The prolonged somatic depolarization that was used 48, 49 would have also increased the size of this hyperpolarizing inhibition by increasing inhibitory drive, as the membrane potential was moved further from the inhibitory reversal potential, and this may explain the absence of a facilitating effect on pyramidal cell spiking. By contrast, the phasic depolarization induced by a dendritic spatial drive from excitatory synapses, in the lingering excitability model, with neurons at resting membrane potential and perisomatic inhibition acting as a shunt [51], may still be sufficient to depolarize pyramidal cells beyond action potential threshold.

Nevertheless, this standalone model cannot explain how goal-directed but not random foraging and/or navigation biases trajectory sequences in awake SWRs to strongly represent the path to the future goal location [37]. Neither does it explain how awake replay occurs in the absence of local sensory drive to place cells. For example, nonlocal forward and backward replay has been observed for trajectories that were either not experienced for more than 10 min [15], or which originated some distance away from the current position of the animal [13]. Moreover, it has been observed that activity in a previous environment is remotely replayed during awake SWRs while the animal is exploring a new environment [53]. Although the first active cell in these remote replays had a higher local firing rate outside of SWRs in the new environment than the last active cell [53], which is consistent with reverse replay depending on the recent firing history of cells [11], this model does not explain how the firing of one initiator cell drives the replay of entire ordered sequences of trajectories from another environment. Clearly, the model also does not explain forward and backward replay during sleep 12, 17, where any residual place selective drive has dissipated.

## Replay as a result of synaptic plasticity

**Figure 2:**
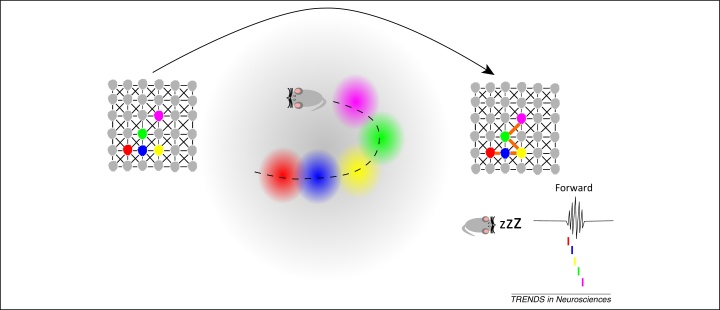
Synaptic plasticity model. In this model, the spatial overlap between the place fields mapping a trajectory in an environment facilitates the strengthening of the excitatory synaptic connections between the transiently co-active place cells. Subsequently, when pyramidal cells are disinhibited during sharp wave ripples (SWRs), the firing of an initiator cell (e.g., the red one) leads to replay of the entire sequence of cells (e.g., the blue, yellow, green, and pink ones) that were previously paired together.

A different model for the generation of sequential replay posits that place cells active during a given trajectory are coupled together by associative synaptic plasticity during exploration. An autoassociative network is required for this model, which may either be provided by the Cornu Ammonis (CA)-3 network alone, or by rapid interactions between CA3 and the dentate gyrus (DG) [54], considering the potential involvement of the DG in promoting SWR activity [55]. Once a given initiator cell in CA3 becomes active during subsequent SWRs, the entire trajectory sequence is reactivated along the path of least resistance, dictated by the internal connectivity and potentiated synapses between cells 9, 12, 46, 56 (Figure 2). The effect of this can then be read out downstream in CA1 through Schaffer collateral connectivity. This can be considered as being similar to how the internal organization of CA3 has been proposed to underlie internally generated theta sequences [57] and preplay activity [40], or indeed how down to up state transitions during the neocortical slow oscillation might initiate spontaneous sequential cortical activity [58]. Since the likelihood of synaptic plasticity is increased following a repeated number of spike pairings, this model would explain how full replay sequences during iSWRs were not visible until at least one, but sometimes several, laps on a track were completed 16, 47. This model is also supported by computational work showing that CA1 pyramidal cell spiking in SWRs is dependent on the strength of their Schaffer collateral connections 59, 60.

If synaptic plasticity is a necessary prerequisite of replay, manipulations that induce plasticity should facilitate SWR replay, while manipulations that prevent plasticity within the hippocampus should not. This prediction has received relatively little support so far, possibly due to methodological considerations, although it has been shown that sSWR-associated unit firing increases following a plasticity-inducing protocol [61]. One approach to blocking plasticity has been to manipulate NMDARs, which are critical for the induction of long-term potentiation at Schaffer collateral and CA3 autoassociational synapses 62, 63. In one study, the NMDAR antagonist CPP was injected before the learning of new goal locations within an already familiar environment [24]. While any synaptic plasticity engaged in encoding the environment would have likely already occurred, the specific reconfiguration of CA1 place cell representations caused by the learning phase [24] would still be liable to perturbation [64]. Consistently, while the mean firing response within eSWRs was not impaired under CPP, the learning-enhanced sleep reactivation of co-activity patterns observed at goal locations was prevented [24]. It would be interesting to know whether the specific blockade of hippocampal NMDARs, rather than systemic CPP injections, has the same effect.

In another study that seemingly challenges the synaptic plasticity model, mice with NMDAR1 knockout (KO) specifically in CA3 pyramidal cells and, therefore, with an absence of NMDAR-dependent long-term potentiation (LTP) at CA3 autoassociational synapses, were found to show stronger, less variable replay of CA1 place cell activity compared with control mice [65]. In this experiment, the mice did not have prior exposure to the environment. During familiarization, lap-by-lap correlations in spiking activity between place cells increased [65], which is a measure of cell assembly formation [66]. This increase and subsequent plateauing was still observed in the KO mice, but at a reduced rate compared with controls [65], not only indicating a role for CA3 synaptic plasticity, but also suggesting that an alternative, potentially plastic, compensatory mechanism, possibly via an alternative autoassociative network, such as DG-CA3, was engaged in binding place cells into coordinated assemblies in these mice. The alternative mechanisms engaged by the NMDAR1 KO mice may have led to the stronger cascading replay activity observed. Therefore, while CA3 NMDAR-dependent synaptic plasticity may not be necessary for the expression of replay *per se*, these studies suggest that NMDAR activity and, as a by product, hippocampal synaptic plasticity, are critically involved in dynamically configuring the hippocampal network into a state that can subsequently bias the SWR activity content. In line with this, while the blockade of NMDARs during learning of new goal locations impaired the sleep reactivation of new place cell representations, it unexpectedly promoted that of old representations [24].

This model is supported by evidence suggesting that reactivation in SWRs is expressed as a function of potentially plasticity-inducing experience. For example, co-activation during awake and sleep SWRs is stronger for cell pairs with overlapping place fields 22, 32, which is a requirement that is critical for the induction of long-term potentiation at Schaffer collateral synapses [67]. Indeed, reactivation in iSWRs improves with experience during exploration [32], in a manner dependent on the repetitiveness of the task and, therefore, the likelihood of place cells to be co-active [68]. Consistently, reactivation during sleep was found to be dependent on the number of times place cells fired together in short windows (<50 ms) during exploration, that is, windows compatible with spike timing-dependent plasticity [56]. Although, since asymmetrical cross-correlations during exploration between cell pairs were not required for reactivation in SWRs, it is debatable whether spike timing-dependent plasticity *per se* is the plasticity mechanism utilized by such a model [32]. By contrast, another carefully designed study found no relation between awake replay and experience. Poorly and extensively experienced trajectories were replayed in similar proportions, never-experienced shortcut sequences were observed during SWRs, and replay was more representative of a scenario independent of experience [15].

This latter observation casts doubt on whether this model alone can sufficiently explain all observable replay phenomena. It is difficult to reconcile a plasticity mechanism that could bind ensembles of cells together in a manner that would enable backward replay preferentially during exploratory behaviors but forward replay during sleep 11, 17, 32. Moreover, the preferential reactivation in SWRs of novel locations or environments over familiar ones 33, 56, 69 (although see [11]), the stronger reactivation on rewarded trials over unrewarded trials [31], and the enhanced reactivation of firing patterns surrounding reward sites 24, 31 are incompatible with the above model when considered in the absence of neuromodulatory drive.

## How does neuromodulation impact SWR activity?

The hippocampus receives constant inputs related to the behavioral state of the animal, including those leading to the release of neuromodulators, which dramatically transform the functional output of neural circuitry [70]. Acetylcholine and dopamine are two such factors whose action within the hippocampus bears particular relevance when considering hippocampal processing during spatial navigation and memory tasks. Here, we propose that these neuromodulatory factors during specific behavioral epochs can explain the observed activity of place cells within SWRs that may otherwise be considered inconsistent with the lingering excitability or synaptic plasticity models when viewed in isolation. Given their different spatiotemporal profiles of release, we propose acetylcholine and dopamine to have different functional roles for the processes underlying memory consolidation. However, at times of simultaneous cholinergic and dopaminergic release, these roles likely occur concomitantly.

### Acetylcholine

Microdialysis measurements of hippocampal acetylcholine levels show variation throughout the sleep–wake cycle, with acetylcholine high during rapid eye movement (REM) sleep and active wakefulness but decreasing levels during quiet wakefulness and SWS [71]. SWRs are generally believed to be initiated when subcortical, particularly cholinergic, drive to the hippocampus is reduced 1, 72. Accordingly, optogenetic stimulation of cholinergic medial septal neurons strongly suppressed SWRs in awake and anaesthetized animals [73], while muscarinic receptor activation suppressed SWRs *in vitro* 74, 75. This could explain why exploratory eSWRs observed during periods of high cholinergic tone occur at a reduced rate compared with awake immobility iSWRs, when the cholinergic tone is reduced [32]. These findings suggest that firing activity during eSWRs and i/sSWRs is differentially modulated by acetylcholine.

Within the hippocampus, acetylcholine exerts wide-ranging cellular and synaptic effects [76]. At the pyramidal cell level, acetylcholine causes membrane depolarization, increased input resistance 77, 78, 79, and enhanced NMDAR currents 80, 81 specifically via the inhibition of SK channels 79, 82. Since somatic depolarization facilitates the emergence of place cell spiking in previously silent CA1 pyramidal cells [83], during eSWRs (but to a lesser extent in iSWRs and not in sSWRs), cholinergic-mediated depolarization would be predicted to facilitate the contribution of subthreshold place-related drive and, thus, the lingering excitability model, to the reactivation of place cell activity. Indeed, place cells have reduced firing rates following pharmacological inactivation of the medial septum 84, 85 or pharmacological blockade of muscarinic receptors [86].

In addition, muscarinic receptor activation by endogenously released acetylcholine *in vivo* 87, 88, or by pharmacological manipulations *in vitro* 79, 89, lowers the threshold for long-term potentiation of excitatory synaptic transmission in the hippocampus. Notably, overlapping CA1 place cell activity was able to engage LTP in CA1 only if sufficient cholinergic tone was present *in vitro* [67]. Therefore, during exploratory activity, acetylcholine likely has a permissive role in the coupling of place-related cell assemblies by synaptic plasticity. It is also possible that the exploration-related cholinergic tone during eSWRs promotes the ability of intra-SWR place cell activity (i.e., replay activity itself) to generate synaptic plasticity. Accordingly, an interesting prediction from this framework is that sSWR activity, while still important for memory consolidation 27, 28, may have a reduced likelihood of inducing plasticity compared with waking SWRs, because cholinergic tone declines. Surprisingly, however, while SWRs have long been posited to provide temporal windows for synaptic plasticity within the hippocampus and in downstream areas 1, 90, few studies have tested whether SWR-driven spiking can induce synaptic plasticity [61].

**Figure 3:**
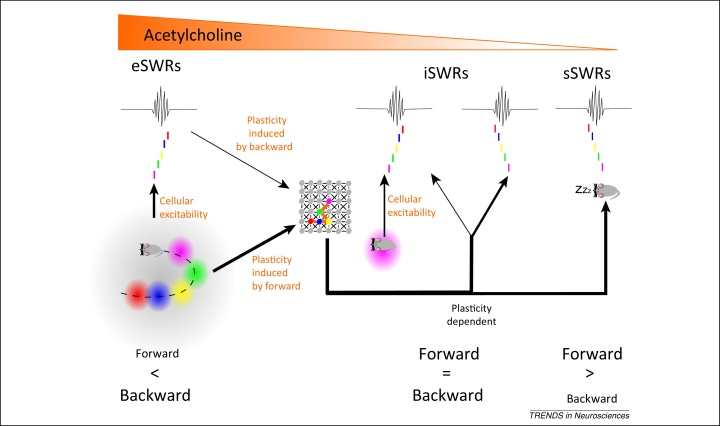
Behavioral state model. During exploration, there are sensory inputs to the hippocampus, place cells are active, and the high cholinergic tone depolarizes cells. These factors all favor predominantly backward replay in exploration sharp wave ripples (eSWRs) 11, 32, in accordance with the model of lingering excitability. It is predicted that this backward replay in eSWRs and the forward activation of place cells during exploration, in the presence of high cholinergic tone, both lead to synaptic plasticity between the active place cells with overlapping place fields [67]. An important assumption is that plasticity induced from environmental exploration is greater than that induced by activity in eSWRs and, therefore, there is a bias in subsequent SWRs for forward replay over backward replay. During longer periods of immobility, this plasticity-dependent forward replay bias is balanced by the lingering excitability of place cells, which, although reduced due to slightly lower levels of acetylcholine [71] and a longer time spent immobile, is still capable of providing an initiation bias to the current position 13, 37, 47 that can drive backward replay. Consequently, during immobility (i)SWRs, there is an equal balance of forward and backward replay [17]. However, since there is a lower level of cholinergic tone, it is predicted that replay during iSWRs induces less plasticity than the replay in eSWRs. Hence, the plasticity-dependent bias for forward replay is maintained. Thus, when the animal sleeps and the lingering excitability and sensory drive to place cells are removed, replay now occurs solely through the plasticity-dependent mechanism and there is more forward than backward replay 12, 17.

Clearly, the impact of acetylcholine on place cell activity during exploration and SWR activity is complex and deserves further investigation by experimentation and computational modeling. Meanwhile, the available literature, outlined above, points towards a model whereby the behavioral state of the animal influences how the hippocampus engages both the lingering excitability and synaptic plasticity mechanisms to initiate replay activity, in a manner strongly shaped by the cholinergic tone (Figure 3). This new framework may go some way to explain the directional bias of replay in different behavioral states 11, 17.

### Dopamine

The hippocampus is also innervated by dopaminergic mesencephalic neurons from the ventral tegmental area (VTA) and substantia nigra [91], although this innervation is sparse [69], with hippocampal dopamine concentrations much lower than in other brain areas, such as the striatum [92]. Notable recent work also suggests that hippocampal dopamine can be released from noradrenergic neurons from the locus coeruleus [93]. VTA neurons exhibit bursting in response to reward or reward-prediction stimuli [94] (reviewed in [95]) and display increased firing, with a higher propensity to fire in bursts, during exposure to novel environments [69]. This is associated with increased dopamine release in downstream areas, including the hippocampus [92].

Interestingly, place cell ensembles are more reactivated in sleep SWRs following the exploration of novel locations and/or environments 33, 56, 69 and following reward-driven learning tasks 24, 31. During SWRs at reward locations, there is also a higher probability of pyramidal cell firing on rewarded versus unrewarded trials [31], and cells with place fields surrounding the reward site have an increased likelihood of firing in both these reward SWRs and subsequent sSWRs 24, 31. Moreover, sequential replay has been shown to be biased towards goal and/or reward sites, with forward and backward replay preferentially representing sequences approaching or ending at the reward site, respectively 15, 37.

Therefore, it is tempting to make the conjecture that dopamine release during exposure to spatial novelty or rewarded outcomes biases the content of subsequent SWR activity for the purpose of memory to represent locations spanning the entire novel environment or the particular behaviorally salient location, respectively. Concordantly, a recent study has shown that burst stimulation of VTA dopaminergic neurons, during exposure to a novel environment (to further enhance novelty-increased VTA firing [69]), subsequently enhanced hippocampal reactivation in a D1/D5 receptor-dependent manner [69]. Neither the general activity of CA1 pyramidal cells during the awake and sleep periods (mean firing rate, SWR-firing rate response, or preferred theta phase) nor the sSWR incidence were modified by such an intervention. These findings suggest that dopamine promotes the consolidation of new memories by the sleep reactivation of newly formed firing patterns. Along this line, it has been further shown that, during the memory retention test of a hippocampal-dependent goal-directed task on a crossword maze, CA1 place maps formed during learning were only partially reinstated and behavioral performance was degraded. However, photostimulation of VTA dopaminergic fibers in dorsal CA1 during learning enhanced SWR reactivation of newly established place cell assemblies. This was accompanied by improved reinstatement of these firing patterns in the retention test and a stable behavioral performance [69]. These findings are consistent with other previous findings showing that midbrain dopaminergic neurons can promote hippocampal place cell dynamics related to memory processing (e.g., 96, 97, 98) and numerous findings providing additional support at the behavioral level (e.g., 99, 100, 101).

**Figure 4:**
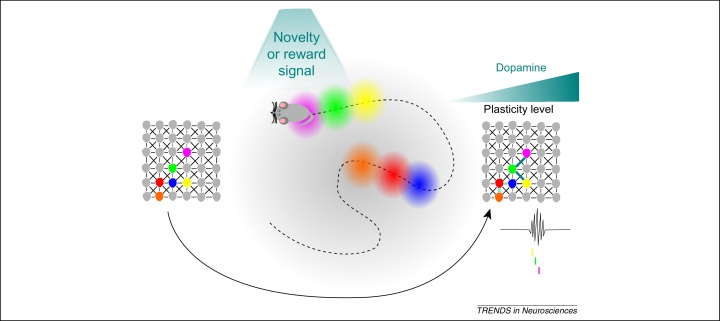
Novelty and/or reward based model. Dopamine release in response to novelty or reward facilitates the formation of stable place cell assemblies through synaptic plasticity (note the stronger connection within the network between the yellow, green, and pink fields compared with the orange, red, and blue fields). This increases the likelihood of replay of cell assemblies active during novel or rewarding environments via the synaptic plasticity model.

It is unclear how dopamine may bias SWR activity through the lingering excitability model since the effects of dopamine on hippocampal pyramidal cell excitability are mixed, with some studies reporting a decrease in excitability, for example by hyperpolarizing the membrane potential and augmenting the spike after hyperpolarization [102], while others report an increase in excitability [103]. However, in both novel environments, and during learning on a goal-driven spatial navigation task, hippocampal place cells remap their activity 8, 24, 104. The stability of these new place cell representations, but not the initial formation, is NMDAR dependent 24, 64 and can be facilitated by D1/D5 receptor activation [98] or optogenetic stimulation of VTA dopaminergic terminals in CA1 [69]. Indeed, pharmacological inhibition of VTA neurons has been shown to impair CA1 place cell stability [97]. These results support a model where dopamine release in novel environments or during reward-driven spatial learning facilitates synaptic plasticity, which then stabilizes place cell activity. Specifically, this may occur via the permissive role of dopamine in the transition from early to late long-term potentiation, potentially through the synaptic tagging and capture hypothesis, as reviewed elsewhere 105, 106. It should also be noted that increased CA1 pyramidal cell firing and changes in CA1 interneuron firing during novel exploration could also contribute to enhanced synaptic plasticity 8, 9, 11, 104, 107. In agreement, novel environment exposure facilitated the induction of LTP at Schaffer collateral synapses to a weak conditioning stimulus *in vivo* and this facilitatory effect was abolished by D1/D5R antagonists or mimicked by D1/D5R agonists [108]. Consequently, via the synaptic plasticity model, dopaminergic modulation likely biases replay to preferentially reactivate cell assemblies relevant to novelty or reward (Figure 4).

How the brain informs the dopaminergic systems about spatial novelty or rewarded outcomes remains to be investigated. This likely involves a complex network of multiple brain circuits 38, 109, 110. For instance, it has been recently shown that VTA dopaminergic cells receive spatiocontextual inputs from the hippocampus via the lateral septum [111]. Moreover, how dopamine release and the firing activity of dopaminergic neurons also relates to aspects of motor actions [112], in addition to reward prediction and spatial novelty, remains to be disentangled.

## Concluding remarks

In conclusion, we have proposed a new conceptual framework for understanding the ordered sequential activation of prior waking activity in SWRs. This likely occurs via a combination of mutually nonexclusive mechanisms, since none of these can explain the available literature in isolation. While many questions remain (Box 3), our current understanding leads us to suggest that the lingering excitability model largely dictates local replaying sequences during awake behavior, while the synaptic plasticity model contributes to subsequent nonlocal awake replays and replaying activity during future rest. Within this framework, we have proposed that cholinergic tone is important for shaping the direction of replay in different behavioral states. Meanwhile, we have identified dopaminergic release during, for example, novel environments and reward-driven spatial tasks, as being important for biasing the content of subsequently replaying trajectories, potentially to strengthen new place cell assemblies and place-reward associations. Testing this new framework experimentally and computationally will be an important step forward for our understanding of the field.

## Figures and Tables

**Figure I:**
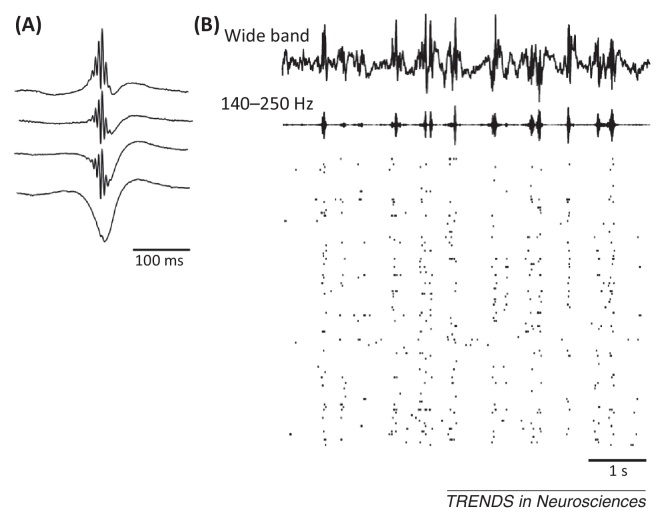
Hippocampal network activity during sleep epochs. **(A)** Mean sharp wave ripple (SWR)-triggered local field potential from four separate tetrodes located just above and within the stratum pyramidale (first three waveforms) and below in the stratum radiatum (bottom waveform). **(B)** SWR firing responses of CA1 pyramidal cells during sleep. Top trace, wide-band (1 H–5 kHz) local field potential recorded in the pyramidal cell layer. Bottom trace, 140–250 Hz band pass-filtered local field potential highlighting ripple frequency events. Raster plots, spike times (vertical tics) of simultaneously recorded CA1 pyramidal cells (one cell per row). Note the firing synchrony during ripple events. Data from [69].

**Figure I:**
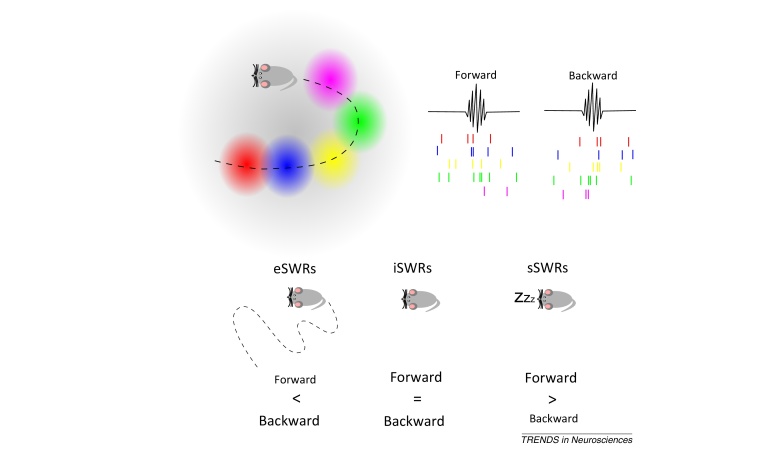
Replay of place cell activity in sharp wave ripples (SWRs).

## Box 1 Sharp wave ripples in the hippocampus

Sharp wave ripples (Figure I) are transient hippocampal network events observed in a variety of species, including humans 43, 113, 114, 115. They comprise negative potentials (sharp waves) in the CA1 stratum radiatum superimposed with short-lived, fast frequency oscillations (ripples) in the CA1 stratum pyramidale. Sharp waves have a current sink in the stratum radiatum of CA1 and CA3 and a current source in the pyramidal cell layer 55, 72, 116, 117, whereas ripples have sink–source pairs at the level of the stratum pyramidale 43, 117. The frequency of the ripple oscillations in CA1 spans a large frequency range [118] that is positively correlated to the amplitude of the sharp wave, with frequencies ranging from 140 to 250 Hz for large sharp waves down to the fast gamma range of 90–140 Hz for smaller sharp waves 55, 119, a frequency that is more akin to those of ripples in CA3 43, 117. Given the absence of CA3–CA1 ripple coherence [117], CA3 units firing in an uncorrelated manner to CA1 ripples [118], incoherent interhemispheric CA1 ripples 43, 120, and decreasing ripple coherence with distance along the septotemporal hippocampal axis [119], ripples are likely to be generated locally within CA1. Indeed, recent work suggests that SWRs occur when a build-up of excitation drives a population of parvalbumin-positive basket cells to fire. Reciprocal inhibition synchronizes this firing at ripple frequency and creates critical windows for alternate basket cell and pyramidal cell firing 121, 122. Approximately 10–20% of hippocampal pyramidal cells fire in any given SWR, but notably usually only with a single action potential each 43, 117.

Despite the lack of widespread ripple coherence, SWRs nevertheless occur simultaneously throughout the hippocampus, subiculum, pre- and parasubiculum cortices, and entorhinal cortex, creating temporal windows of heightened neuronal firing 120, 123 and widespread changes in activity throughout the cortical network [124]. As such, they have been proposed as an ideal candidate to transfer labile hippocampal memories to more stable neocortical sites during offline states 72, 90 and also to downstream structures, including the ventral striatum 125, 126.

## Box 2 Replay of place cell activity

As a rodent follows a spatial trajectory, place cells are sequentially activated. In subsequent SWRs, this sequence of place cells may be reactivated in a time-compressed manner in either a forward or a backward direction. The directional bias is dependent on the behavioral state of the animal, with more backward replay in eSWRs, a similar occurrence probability of forward and backward replay in iSWRs, and more forward replay in sSWRs 11, 17 (Figure I).

## Box 3 Outstanding questions

• The ability of replay activity to drive excitatory synaptic plasticity within the hippocampus and between the hippocampus and neocortical sites, a central concept to the proposed function of SWRs in memory consolidation, has received limited experimental attention [61]. Can *in vivo* patterns of SWR-related firing drive excitatory synaptic plasticity *in vitro* at specific synapses? How does cholinergic tone impact how SWR activity induces synaptic plasticity?
• Similarly, evidence testing whether synaptic plasticity can drive pyramidal cell firing in SWRs is limited [61]. By artificially inducing synaptic plasticity onto specific cells and within cell assemblies, can the content of SWR activity be biased? What type of synaptic plasticity is required and at which synapses?
• Given the role of inhibition in limiting pyramidal cell spiking in SWRs, how might activity-dependent interneuron plasticity influence SWR pyramidal cell spiking? Could this provide another mechanism for selecting which place cell ensembles are replayed?
• We have assumed that the action of acetylcholine on membrane depolarization and input resistance can facilitate the contribution of subthreshold place-related drive and, thus, the lingering excitability model, to the reactivation of place cell activity. This could be tested similarly to [83] by asking whether optogenetic stimulation of cholinergic input to the hippocampus can determine the emergence of new place cell representations in previously silent CA1 pyramidal cells.
• The behavioral state model could be tested computationally by incorporating rules governing how cholinergic tone influences spiking and synaptic plasticity (both by theta activity and SWR activity) into a model of forward and backward replay during SWRs (e.g., 128, 129). The model could then be used to ask whether cholinergic input could explain the transition in replay direction preference between eSWRs, iSWRs, and sSWRs.
• We have proposed that dopamine release during exploration biases subsequently replaying trajectories via the synaptic plasticity model. Our behavioral state model predicts that this could bias forward over backward replay when dopamine is present. This could be tested by assessing the replay direction of place cell activity at a reward site on rewarded versus unrewarded trials. In addition, can physiological optogenetic stimulation of dopaminergic terminals bias the content of replay to represent an artificial and/or pseudo-reward site?
• Given the involvement of the DG in SWR activity [55] and the influence of the spatially tuned entorhinal cells (e.g., grid and boundary cells) on place cell activity [130], how might activity in the DG and entorhinal cortex influence replay activity?

## Glossary

Acetylcholine: a neuromodulator and neurotransmitter with numerous functions, including attention, learning, arousal, and synaptic plasticity, which mainly exerts its actions via nicotinic and muscarinic receptors.
Current sinks and sources: subdomains along the neuronal membrane where net positive charge flows into (sinks) or out of (sources) neurons. The location of sinks and sources is inverted for a net negative charge.
Dopamine: a neuromodulator classically implicated in reward or reward-prediction error; it exerts its actions via G-protein-coupled receptors.
Hippocampus: a structure within the medial temporal lobe that is important for episodic memory, spatial learning, and associational recollection. It comprises CA1–3 and the DG. Input to the hippocampus comes primarily from the entorhinal cortex via axons of the perforant pathway and temporoammonic pathway, which connect the entorhinal cortex with the DG and CA3, or CA1 respectively. Hippocampal mossy fibers are axons connecting the DG to CA3, while axons of the Schaffer collateral pathway connect CA3 to CA1. CA1 axons then project out of the hippocampus to the subiculum.
Medial septum/basal forebrain: primary source of cholinergic fibers innervating the neocortex and hippocampus.
Place cells: hippocampal principal cells increasing their discharge of action potentials in a specific location (place field) of the environment.
Reactivation/replay: the reoccurrence during off-line states of the firing patterns of hippocampal principal cells previously observed during active waking behavior. These waking activity patterns can either represent discrete places or extended sequences of place cell activity.
Sharp wave ripples (SWRs): hippocampal transient network events manifest as negative potentials (sharp waves) in the CA1 stratum radiatum superimposed with short-lived, fast (140–250 Hz) frequency oscillations (ripples) in the CA1 stratum pyramidale. SWRs mainly occur during long periods of behavioral awake immobility and SWS.
Synaptic plasticity: an activity-dependent change in the efficiency of synapses. If occurring for an extended period of time, an increase in synaptic strength is referred to as long-term potentiation (LTP), while a decrease is referred to as long-term depression (LTD).
Theta phase precession: the phenomenon whereby a place cell spikes at progressively earlier phases of a theta cycle during movement through the place field of that cell.
Ventral tegmental Area (VTA): midbrain structure containing the dopaminergic cell bodies of the mesocorticolimbic dopamine system. The VTA targets a large number of structures, including not only the accumbens, olfactory tubercle, orbitofrontal cortex, motor cortex, striatum, lateral septum, ventral pallidum, extended amygdala, subventricular zone, and lateral habenula, but also the entorhinal cortex and hippocampus.
